# Digital Image Correlation Comparison of Damaged and Undamaged Aeronautical CFRPs During Compression Tests

**DOI:** 10.3390/ma12020249

**Published:** 2019-01-13

**Authors:** Claudia Barile, Caterina Casavola, Giovanni Pappalettera

**Affiliations:** 1 Department of Mechanics, Mathematics and Management, Politecnico di Bari, viale Japigia, 70126 Bari, Italy; casavola@poliba.it (C.C.); giovanni.pappalettera@poliba.it (G.P.); 2 IMAST S.c.a.r.l., Distretto Tecnologico Per L’ingegneria Dei Materiali Polimerici E Compositi E Strutture, P.zza Bovio 22, 80133 Napoli, Italy

**Keywords:** Carbon Fibre Reinforced Plastic Materials (CFRP), compression tests, Digital Image Correlation (DIC), buckling

## Abstract

The diffusion of composite materials in aeronautical and aerospace applications is attributable to the high specific mechanical properties they offer. In particular, the recent use of Carbon Fiber Reinforced Polymer (CFRP) materials is highly increased. The main disadvantage in using this kind of material is related to the possibility of including damages or defects not visible on the surface that compromise their behavior and make their use extremely unsafe if not properly supervised. The most conventional nondestructive techniques allow the detection of damages when they already compromise the life of these materials. The use of the same techniques makes it harder to monitor in-situ of the progress of damages, especially if they occur inside the materials. The implementation of the innovative strain analysis method, like those based on full-field measurements, could provide additional information about the damage mechanisms by supplying the complete strain distribution of the surface of the sample. The present paper examines the mechanical behavior of two different CFRP specimens, with and without damage, subjected to compressive load in an anti-buckling fixture by using the Digital Image Correlation (DIC). The purpose is to measure the out-of-plane displacements, characteristics of the compression tests, in all the points of the ROI (Region of Interest), using a full-field and noncontact technique. The innovative aspect of this work is therefore to solve this problem through an experimental approach with DIC 3D technique.

## 1. Introduction

Composite materials are carried out by combining two or more materials with dissimilar mechanical and physical properties. Provided the matching is well done, the final material will have better overall properties if compared to the parents. A well-consolidated class of composite materials is obtained by using a matrix material combined with a reinforcing one. In this way, it is possible to obtain a continuous material with increased capability of loading transmission and higher resistance. From a classification point of view composites can be considered anisotropic, nonhomogeneous materials, in which each of the constituting materials is inextricably connected with the others. Carbon Fiber Reinforced Plastics (CFRP) is one of the most interesting composite class of materials and it will be specifically analyzed in this paper.

The great interest in CFRPs is driven mainly by their valuable mechanical properties [1,2,3]. In fact, they combine high stiffness and high strength to low density, low crack sensitivity, and good properties under fatigue loading. At the same time, they are appealing for applications where vibration dumping is required [4]. Another point of strength of these materials is connected with their high-dimensional stability [5]. In fact, due to the low thermal expansion coefficient, they allow the obtainment of stable structures, even if a large range of exercise temperatures must be taken into account.

Epoxy resins are typical materials for obtaining the matrix [6]. This is a family of thermosetting polymers quite interesting because of their low coefficient of reticulation retention and the absence of sub-product consequent to the curing process. Moreover, epoxy resins are characterized by good mechanical properties, good environmental resistance, good adhesion to many different materials, and a low coefficient of thermal conductivity [7]. In addition, it is worth noting that this material is characterized by a considerable strength-to-density ratio (SWR). This property, in particular, makes this kind of material of great interest in the aeronautical and automotive fields and, more in general, wherever lightweight of structure is a crucial point in the designing process. 

Due to the fact that CFRP are, in general, nonhomogeneous and anisotropic, the mechanical response of these materials is dependent on the relative orientation of the applied load with respect to the fibers. Traditional approaches of experimental characterization can fail in capturing the complex behavior of such a kind of materials, so that modern approaches based on full-field optical methods [8,9,10,11] successfully employed in other applications where nonhomogeneous materials are analyzed [12,13,14,15] can be used for CFRP, as will be illustrated in this paper. More recently, approaches based on Digital Image Correlation (DIC) were applied to these kinds of problems [16,17,18]. It worth noting that the possibility to have a high-resolution full description of the behavior of the component opens the possibility to make numerical modelling more accurate and refined, so to improve and extend capabilities of the whole design process [19,20].

In this work, in particular, the stereo DIC technique was implemented. In this technique, a random speckle pattern is applied to the surface of the component under testing. During the test, the variation of the speckle pattern is followed by two CCD (Charge-Coupled Device) cameras observing the sample from two different directions. Correlation of the speckle pattern at each stage of the test allows obtaining the full 3D displacement field (x,y,z) for each of the point in the region of interest [21]. In this way, it is possible to determine to in-plane strain field, as well as the out of plane displacement component that occurs, for example, in presence of buckling phenomena.

In this work two types of CFRPs are analyzed: One is a laminate made up by multi-plies CFRP, the second is a foam sandwich included in two sheets of CFRP, the skin. Specimens of the two materials were subjected to compression load, as described by the Compression After Impact (CAI) standard [22]. According to the aeronautical requirements, and to better describe the response of both materials, two types of initial conditions of the specimens were considered: As delivered, and with an impacted damage [23]. Three specimens per type and condition were tested.

## 2. Materials and Methods 

### 2.1. Material Description and Preparation for Measurements

In this study, two diverse types of CFRP were analyzed. The first is a laminate made by multi-plies epoxy resin reinforced by carbon fibers. The second is a sandwich constituted by a foam included in two skins of CFRP. In both cases the specimens were tested under uniaxial compression conditions, according to the CAI Test (ASTM 7137) [22]. According to the standard, the width and the height of both the materials’ specimens were 150 mm × 100 mm, while the thicknesses were, respectively, 5 mm for the laminates and 3 mm for the foams.

According to the producer’s reference, the layout of the laminate’s plies is reported following:

- [45F/0T/0T/45F/0T/0T/0F/0T/0T/0F/0T/0T/45F/0T]_s_ where F = Fabric, T = Tape.

On the other hand, the layout of the foam sandwich is indicated following:

- [45F/0F/FOAM 3mm/0F/45F] where F= Fabric.

Specimens of both materials were tested in compression as delivered and after the impact tests. 

**Figure 1:**
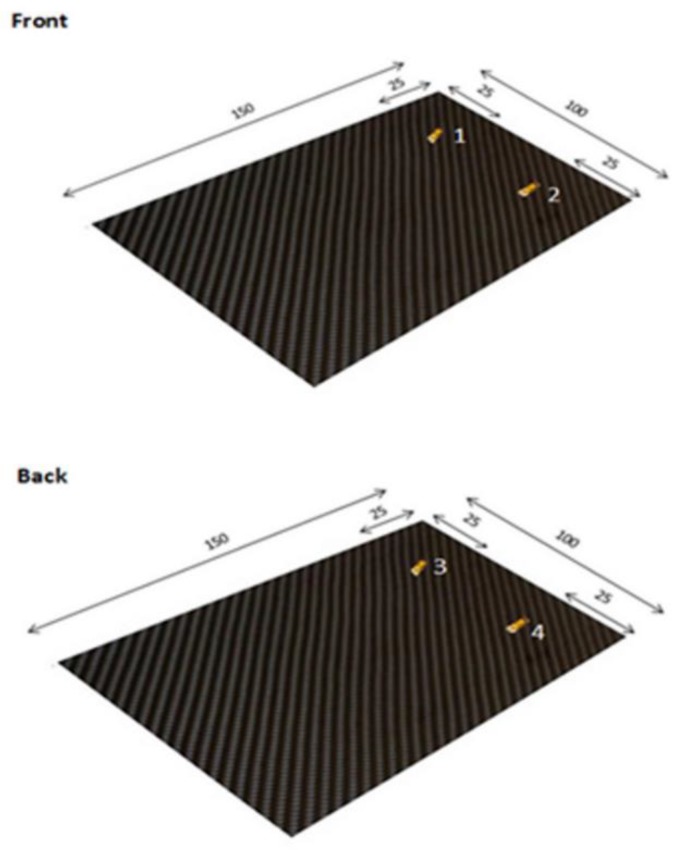
Strain gages application on composite materials [dimensions in mm].

According to the standard, the evaluation of the local strain was obtained by applying four electrical strain gauges (Figure 1), two for each side of the samples [22]. 

**Figure 2:**
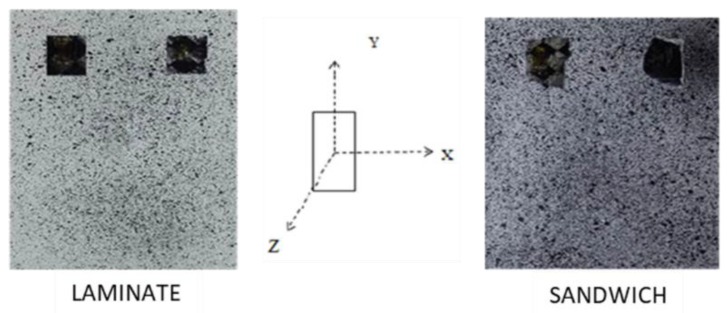
Real components sprayed for Digital Image Correlation (DIC) analysis.

In order to allow strain field analysis by DIC, a random speckle pattern was preliminarily painted over the surface of the samples to be analyzed. This was done by spraying the specimen with a white matte spray to obtain a uniform white background and, successively, by spraying black speckles on the surface (Figure 2).

The aim of this approach is to compare the local behavior of materials recorded by the strain gauges with the overall behavior obtained by DIC.

Compression test were made by following the standard ASTM 7137 [22]. Tests were performed at 1.25 mm/min displacement rate on a SCHENCK servo-hydraulic machine with a 250 kN load cell. Initially, positioning of the specimen was done with great care to avoid the presence of torsional and bending loads.

### 2.2. Configuration of the Measurement Chain

**Figure 3:**
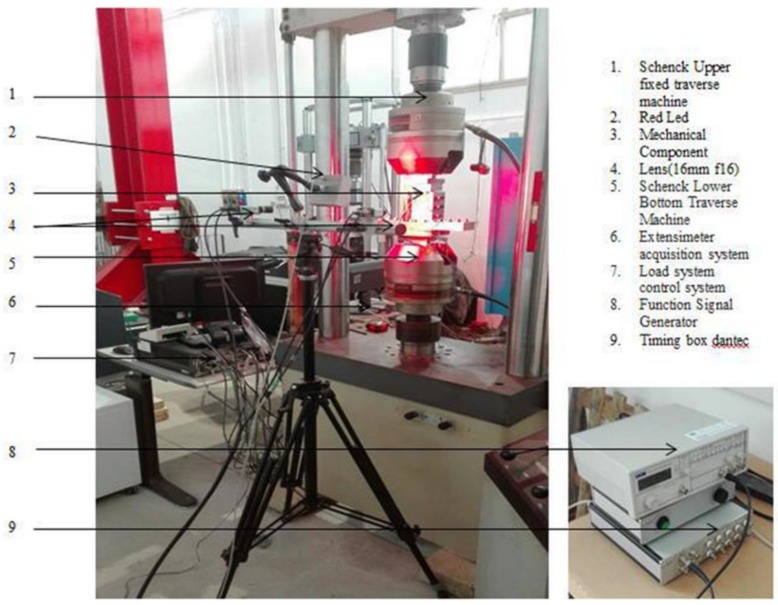
Overview of experimental set-up.

The measurement of displacements and deformation on the entire surface of the sample was obtained by means of the DIC system Dantec Dynamics Q400 (Dantec Dynamics A/S, Skovlunde, Denmark). It is equipped with ISTRA 4D software (Dantec Dynamics A/S, Skovlunde, Denmark), and it includes two 1628 × 1436 pixels GigE CCD Manta by AVT (Allied Vision Technologies GmbH, Stadtroda, Germany) industrial cameras, equipped with Ricoh 16 mm lenses based the extensions of the area under analysis. The two cameras are mounted on the same support bar in order to compensate vibrations. Image acquisition is triggered by a synchronization unit including National Instruments^®^ NIDAQ 9171 acquisition card (National Instruments Italy S.r.l., Assago, Italy), which converts the acquired signal analog to digital. The synchronization units are connected both with the loading machine and a PC dedicated to the system management. The starting of the acquisition was synchronized with the starting of the test by using an output TTL (Transistor-Transistor Logic) signal from the loading machine. The signal was active as soon as the test started and was used to trigger the synchronization unit. A frequency of 1 Hz was set for the tests. A led source is used to stably and evenly illuminate the area to be analyzed. By using this kind of illumination, higher intensity stability, in time, is achieved. This results in a more stable correlation process. Initial tuning of the illumination level was performed in order to optimize the acquisition by avoiding both saturation and low illuminated pixels in the whole object area. An optic with a small focal length was chosen due to the possibility of capturing suitably large test specimens and to monitor the whole component (Figure 3).

Settings for the DIC apparatus are as follows:

- Span length between cameras 40 cm
- Object’s distance from the cameras 50 cm
- Cameras’ Inclination 21.8°
- Diaphragm opening f/8

**Figure 4:**
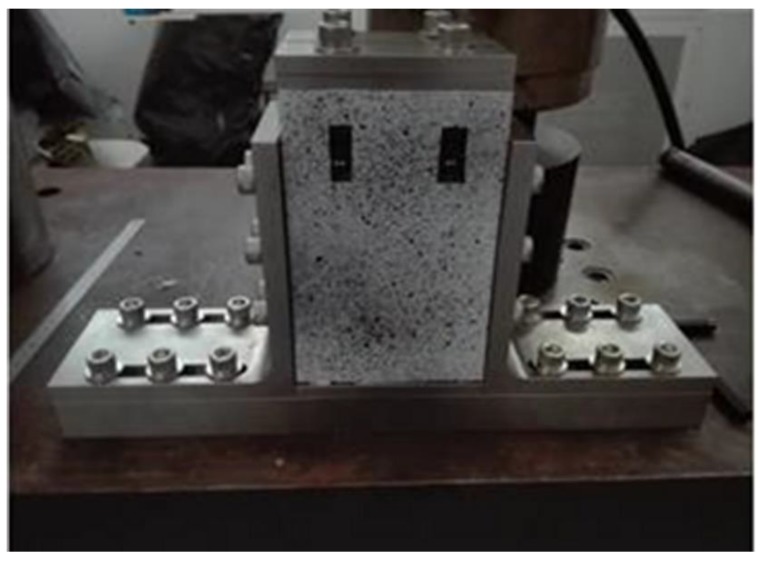
Edge-constraint structure adopted in the experiment.

The compressive fixture used for the anti-buckling tests is shown in Figure 4. It includes adaptable retaining plates (base and top plates, and two slide plates) for supporting the specimen and prevent buckling during the load application. The fixture allows regulation for considering small variations in specimen sizes. The side plates are shorter than the specimens to ensure the proper sample absorption of the load. The configuration of the features could deeply affect the test results. In the standard test fixture, the base and the top supports provide no clamping but only some restraints to local out-of-plane rotation. 

**Figure 5:**
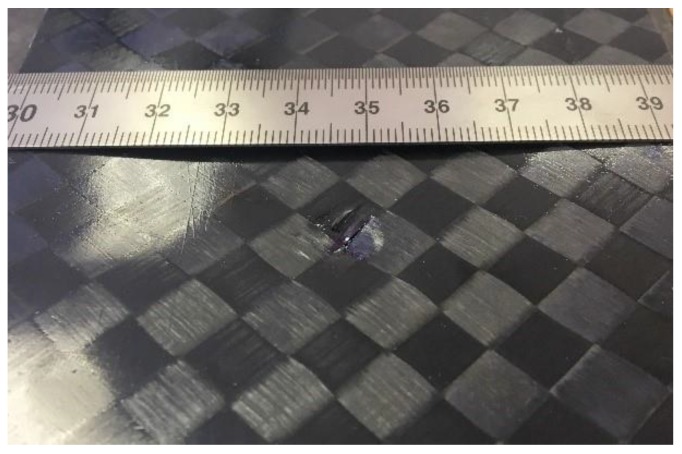
Shape of the damage created on each specimen.

According to the standard [22], the specimens used for that kind of test were previously subjected to drop-weight impact per Test Method D7136 [23] prior to application of compressive force. As prescribed by the standard, the specimen was placed on a fixture base and aligned with the dropping axis by using guiding pins. Once finished, the alignment operations the specimen was clamped by four rubber terminated toggles. Velocity of the impactor was determined by optical sensors (manufacturer, city, country) placed along the dropping axis to determine accurately the impact energy. Experiments were led at room temperature at two levels of energy, respectively, 50 J for CFRP and 10 J for foams. Impact was obtained by a hemispherical impactor (manufacturer, city, country) having 16 mm of diameter, as prescribed by the standard. The rebound catcher system was activated during the test to avoid rebound of the impactor on the tested sample. These drop tests are most used in the aeronautic field for simulating the impact of a structure with birds. With the new composite materials, this aspect is of great interest for industry in order to evaluate the delamination effects occurring. Moreover, they generate a damage on the specimens whose shape and dimensions depend on the energy level of the test, as well as on the structural lay-up of the materials. According to the producer’s requirements, the above indicated energy levels set for the two materials were selected, after preliminary tests, to ensure a Barely Visible Impact Damage (BVID) of about 1 mm in depth (Figure 5). The extension of the damaged area was 1 cm for both the analyzed class of materials. The BVID is the name given to damage in a composite material caused by a low-velocity impact from objects. Low-velocity impacts cause only minor damage to the surface of the composite, which is generally not readily detectable by visual inspection, hence the term BVID. However, they can cause structurally considerable damage internally within laminate composites, such as delamination, matrix cracking, fiber fracture, and fiber pullout. The aim of the CAI tests was to evaluate the compression residual strength properties of CFRP. 

The 3D system had acquired a series of images representing the displacement off the plane (z-displacement). It recorded, for the entire duration of the test, all the configurations of the component from the first undeformed one to the break occurrence.

## 3. Results and Discussion

**Figure 6:**
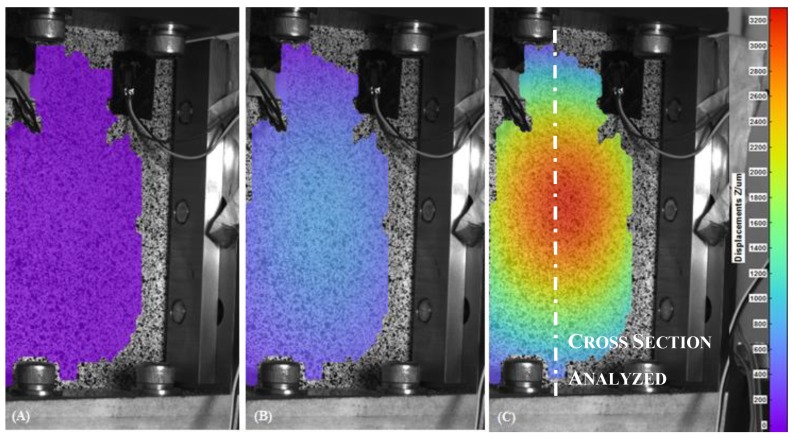
Out-of-plane displacement of the undamaged Carbon Fiber Reinforced Plastics (CFRP) recorded by DIC at t = 0 s (**A**), t = 80 s (**B**) and t = 116 s (**C**).

**Figure 7:**
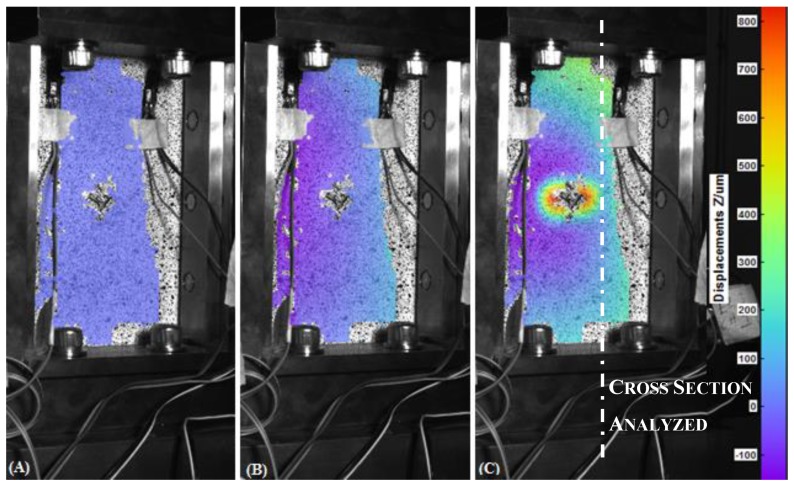
Out-of-plane displacement of the damaged CFRP recorded by DIC at t = 0 s (**A**), t = 60 s (**B**) and t = 101 s (**C**).

**Figure 8:**
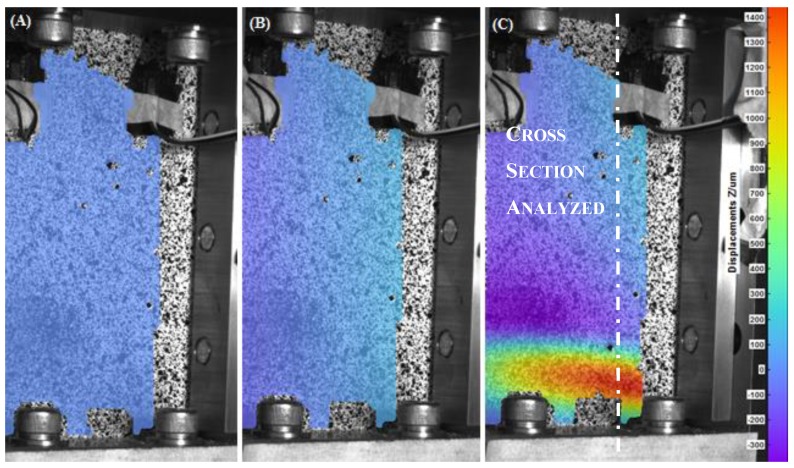
Out-of-plane displacement of the undamaged foam recorded by DIC at t = 0 s (**A**), t = 45 s (**B**), t = 60 s (**C**).

**Figure 9:**
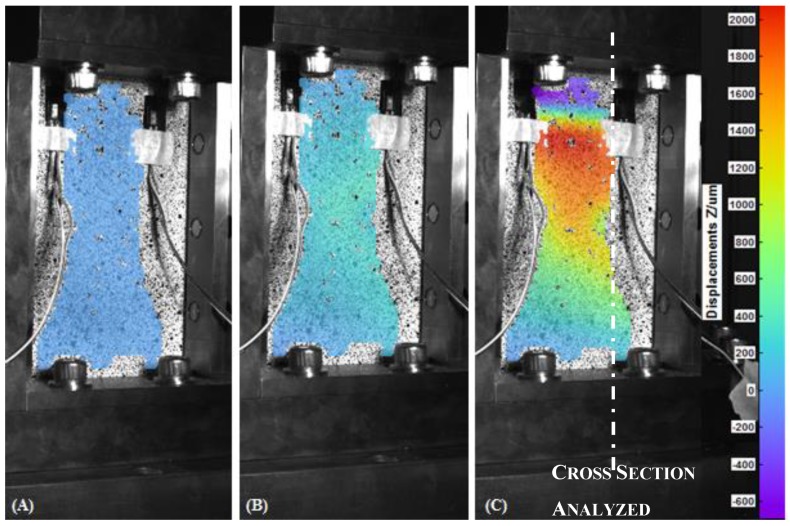
Out-of-plane displacement of the damaged Foam recorded by DIC at t = 0 s (**A**), t = 55s (**B**) and t = 70 s (**C**).

In Figure 6, Figure 7, Figure 8 and Figure 9, the out-of-plane displacement field obtained at different instant times during the uniaxial compression tests on CFRPs and foams, damaged and undamaged, are shown. Main analysis settings for this evaluation are listed as follows: 3D residuum lower that 0.4 pixels, facets size 19 pixels, and accuracy 0.1 pixels [16]. A total of 1180 grid points are computed on average in the ROI.

Referring to the laminate specimens and from the analysis of the out-of-plane displacements, several considerations can be done. First, it can be observed that, both the fixture and the low displacement rate (1.25 mm/min) contribute to keep low the amount of the out-of-plane displacement in the initial part of the test, for both the CFRP and foam components.

**Figure 10:**
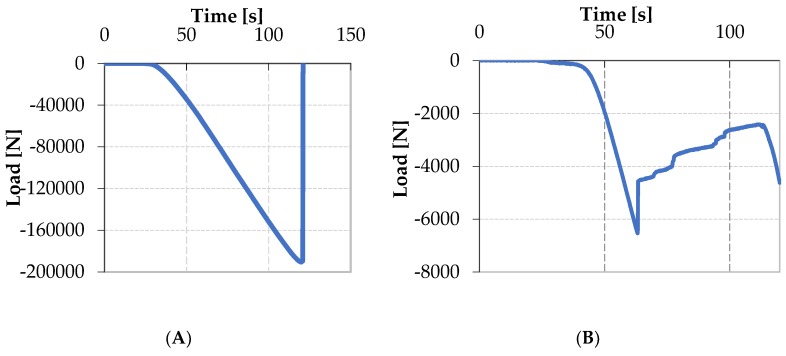
Load law evolution of the machine for the CFRP specimen (**A**) and foam composite (**B**).

Initial settlement of the loading plate is observable in the first 20 s of the test, as shown in Figure 10A. From that point, on load starts to be effectively transmitted to the sample. The CFRP laminates begin to collapse after 80 s (inflexion point) from the start of the test, when an out-of-plane displacement of 800 μm is clearly recorded by the DIC system. After 116 s, out-of-plane displacements become more evident and they reach a maximum value of 3200 μm, while the specimen broke soon after, namely at 120 s. The abrupt cut of curve is caused by the failure of the component. This happened for a load equal to −200 kN. 

Similar analysis, but different considerations, can be done for the foam specimens, as shown in Figure 10B. In this case, an initial stage of adjustment of the loading fixture is observed. Starting from t = 45 s, load is effectively transmitted to the specimen and a linear trend is followed until a rapid collapse of the specimens occurred at t = 63 s, when the applied load reached a value of −6.6 kN. This value corresponds to the maximum resistance of the face sheets of the sandwich. After that, differently from the previous reported case load is still recorded as a consequence of the presence of foam which is, in this stage, still capable to transmit load. Load starts to increase again at time t = 122 s due to the reached contact between the upper and the lower part of the fixture. It should be observed that, in this situation, appearance of instability is not directly inferable by the temporal load law.

High-definition maps of quantitative performance of out-of-plane displacements collected by means of the DIC technique allow an immediate understanding of the global and local behavior of the components, before and after the damage, highlighting the different responses of the materials investigated.

Next, a series of plots reporting the normal strain along y, the shear strain and the displacement along z for CFRP and foam, respectively, will be presented. They are representative of what obtained on the three replications of each combination of material/damage condition.

**Figure 11:**
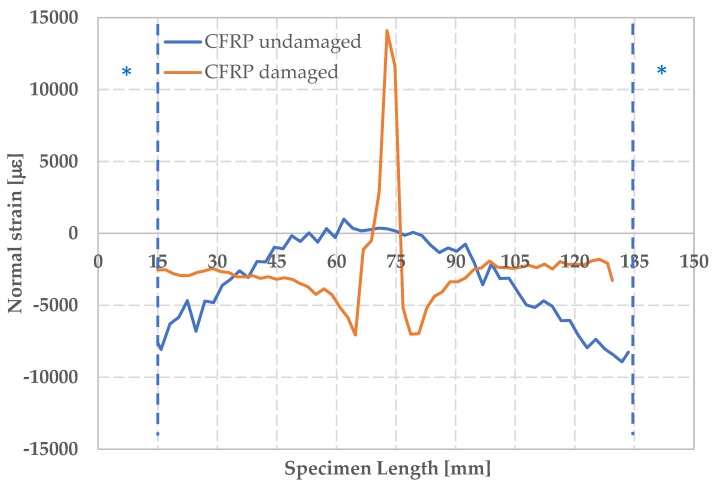
Maximum normal strain ε_yy_ for damaged and undamaged CFRP.

The longitudinal deformation ε_yy_ along the median cross section of the CFRP specimens is shown in Figure 11, under conditions of maximum deformation. Cross-section was chosen in order to avoid regions where no correlation was observed as shown in Figure 7. The * indicates areas with no information due to the impossibility of recognizing speckle pattern by the DIC system. The lack of information is caused by the shadow zones created by the fixture.

A different behavior is clearly observable for the damaged and the undamaged components: the mean values of maximum load are −102,714 ± 8286 N and −192,674 ± 10,081 N, respectively, while the mean values of maximum CAI stress are −202 ± 15 MPa and −385 ± 20 MPa, respectively. The undamaged specimen displays a normal strain negative in the outlying areas, showing a symmetrical shape with respect to the middle part of the component. This is in accordance with what was expected for undamaged component under fixed-fixed column constraints conditions. The damaged composite undergoes a more evident buckling deformation in the center of its structure.

**Figure 12:**
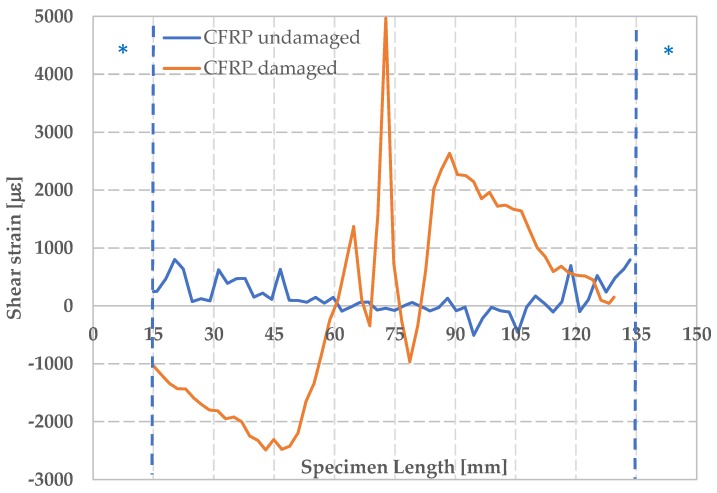
Maximum shear strain ε_xy_ for damaged and undamaged CFRP.

In Figure 12, the maximum shear strain for damaged and undamaged CFRPs is also reported. No points of maximum shear strain concentration, along the analyzed cross-sections, are observable for the undamaged CFRPs. For the damaged CFRPs, instead, a very different behavior is observed with a clearly evident maximum at the center of the component with a positive strain of 5000 μm. Rupture of the component occurs in correspondence of the area of maximum shear strain. A symmetric behavior in the lower part of the sample with respect to the upper part is observed, indicating the presence of a relative rotation of the two parts during the test. 

**Figure 13:**
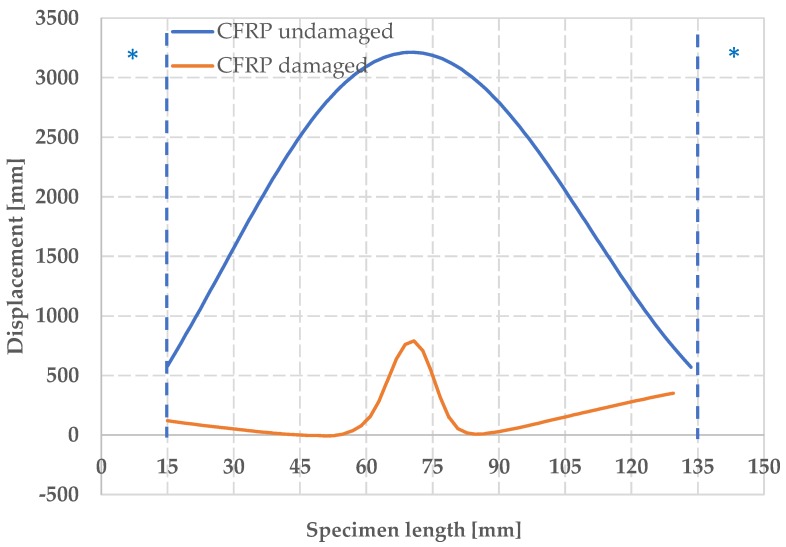
Out-of-plane displacement w for damaged and undamaged CFRP.

The off-plane displacement behavior allows to localize the buckling (Figure 13). For the damaged CFRPs, it results located exactly at the center, reaching a value w equal to 750 µm at 75 mm along the y axis, and a value of w equal to 3200 µm for the undamaged CFRP.

**Figure 14:**
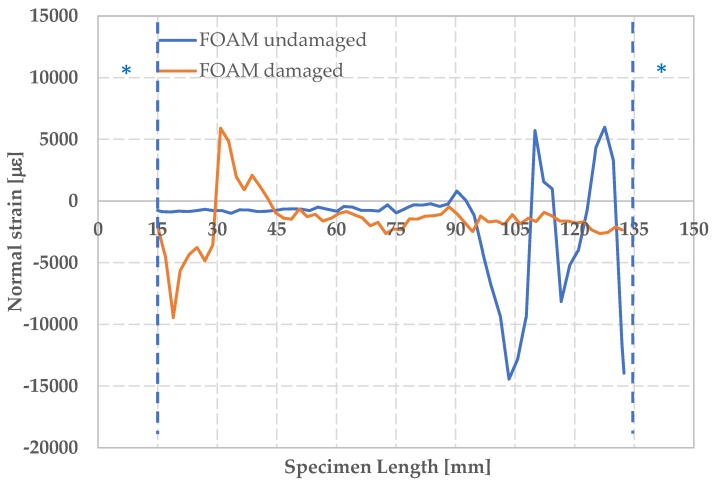
Maximum normal strain ε_yy_ for damaged and undamaged foam.

In Figure 14, the trend of longitudinal deformation along the cross-section for the foam specimens is reported under conditions of maximum longitudinal deformation. The maximum values of ε_yy_ along the median cross section are, respectively, −8000 με for the undamaged foam and −9700 με for the impacted foam. The correspondent mean values of maximum load are −6618 ± 206 N and −4178 ± 413 N, respectively, while the mean values of maximum CAI stress are −84 ± 3 MPa and −53 ± 5 MPa, respectively.

The maximum deformations of the component are not recorded at the center. It is noticed that the not impacted specimens buckle in the lower part while the impacted foams reached the buckling in the upper part. 

**Figure 15:**
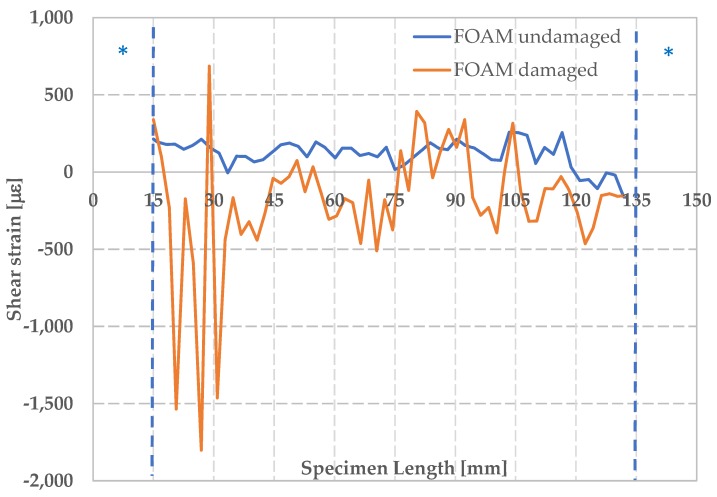
Maximum shear strain γ_xy_ for damaged and undamaged foam.

In Figure 15, the maximum shear strain recorded under conditions of maximum deformation is shown. It can be observed that the shear strain, for the undamaged foam, is quite uniform along the plotted cross section. In the case of damaged foam, we can observe points of shear strain concentration along the analyzed cross section; in particular high negative values of shear strain are recorded in correspondence of the upper part of the component reaching a minimum value of γ_xy_ of −1800 μm, that corresponds to the region where skin instability occurs.

**Figure 16:**
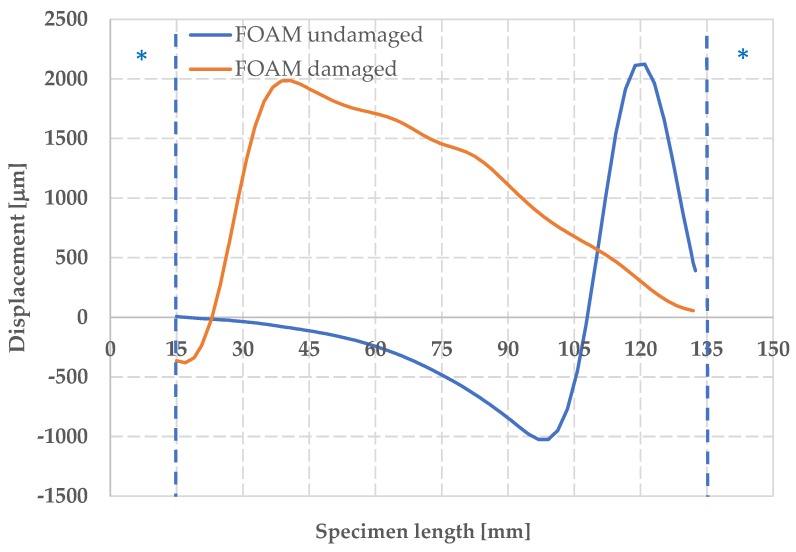
Maximum out-of-plane displacement w for damaged and undamaged foam.

For the undamaged foam, the graph of the out-of-plane displacement (Figure 16) shows a maximum value of w equal to 1400 μm in correspondence of a length of 120 mm, just before the rupture at t = 62 s.

In the damaged foam, a globally higher out-of-plane displacement field is observed, reaching a maximum of w = 2000 μm in the upper area of the specimen.

It is worth noting that, for all reported data, the phenomenon of elastic instability occurs even if the specimens were constrained by fixture properly designed to avoid instability. However, the critical load is higher than that referenced by the bibliography for a simply anisotropic supported plate subject to uniaxial compression [24]. Moreover, it worth adding some additional general considerations: First, presented analysis indicates that analysis of out-of-plane displacement is an additional indicator to evaluate the presence or appearance of damage and this can be flanked to normal and shear strain that were used in experiments on CFRP by Reference [25]. This is obtained by also taking into consideration that a buckling minimizing clamping system was implemented for performing the test. Strikingly, capability of information provided by DIC to put in evidence the presence of damage is here confirmed also by the presence of a real impact damage while similar evidence was previously found for the case of a simulated damage by a drilled and repaired hole [25]. Another important aspect to put into evidence is the very different behavior under compression shown by the CFRP and foam, respectively. While in the first case, by analyzing a cross-section, strain concentration points appear in the proximity of the damage, in the case of the foams a more homogenous behavior appears with a strain concentration points that are shifted away from the center of the damage. This is a behavior different from what observed by Reference [26] where, however, a higher impact energy was used.

## 4. Conclusions

In this paper, the mechanical behavior of composites subjected to compression loading was analyzed by DIC. By the 3D DIC, high-resolution maps were obtained, reporting quantitative trends of the out-of-plane displacements of the investigated surfaces at different loads. These maps allowed an immediate understanding of the global and local behavior of the components, highlighting the different responses of the investigated materials. When those maps are used to compare mechanical behavior of damaged and undamaged components, they show how the presence of the impact damage drastically change the response of the component.

Interesting consideration was found concerning with the measurement of the shear strain γ_xy_ before the break of the specimens. In fact, it was found that, for impacted specimens, both CFRPs and foams, the fracture point can be identified in correspondence of the absolute maximum values of γ_xy_. Similar consideration cannot be extended to undamaged specimen. This aspect of the DIC is significant as it allows to predict the area of component breakage. Potentialities of DIC technique are evident also in relationship with the capability of observing occurrence of instability during loading. It was found that buckling can be hardly be inferred by observing the temporal load law for the CFRPs while no evidence appears when the law is analyzed for foams. DIC, instead, clearly allows to detect buckling as well as to identify its localization over the component.
